# Phase III clinical trial of autologous CD34 + cell transplantation to accelerate fracture nonunion repair

**DOI:** 10.1186/s12916-023-03088-y

**Published:** 2023-10-05

**Authors:** Ryosuke Kuroda, Takahiro Niikura, Tomoyuki Matsumoto, Tomoaki Fukui, Keisuke Oe, Yutaka Mifune, Hironobu Minami, Hiroshi Matsuoka, Kimikazu Yakushijin, Yoshiharu Miyata, Shinichiro Kawamoto, Tatsuo Kagimura, Yasuyuki Fujita, Atsuhiko Kawamoto

**Affiliations:** 1 Department of Orthopaedic Surgery, Kobe University Graduate School of Medicine, 7-5-1 Kusunoki-Cho, Chuo-Ku, Kobe, 650-0017 Japan; 2 Division of Medical Oncology/Hematology, Department of Medicine, Kobe University Hospital and Graduate School of Medicine, Chuo-Ku, Kobe, 650-0017 Japan; 3 Department of Transfusion Medicine and Cell Therapy, Kobe University Hospital, Chuo-Ku, Kobe, 650-0017 Japan; 4 Translational Research Center for Medical Innovation, Foundation for Biomedical Research and Innovation at Kobe, Chuo-Ku, Kobe, 650-0047 Japan

**Keywords:** Clinical trial, Phase III, CD34 + cell, Transplantation, Fracture nonunion

## Abstract

**Background:**

We previously demonstrated that CD34 + cell transplantation in animals healed intractable fractures via osteogenesis and vasculogenesis; we also demonstrated the safety and efficacy of this cell therapy in an earlier phase I/II clinical trial conducted on seven patients with fracture nonunion. Herein, we present the results of a phase III clinical trial conducted to confirm the results of the previous phase studies using a larger cohort of patients.

**Methods:**

CD34 + cells were mobilized via administration of granulocyte colony-stimulating factor, harvested using leukapheresis, and isolated using magnetic cell sorting. Autologous CD34 + cells were transplanted in 15 patients with tibia nonunion and 10 patients with femur nonunion, who were followed up for 52 weeks post transplantation. The main outcome was a reduction in time to heal the tibia in nonunion patients compared with that in historical control patients. We calculated the required number of patients as 15 based on the results of the phase I/II study. An independent data monitoring committee performed the radiographic assessments. Adverse events and medical device failures were recorded.

**Results:**

All fractures healed during the study period. The time to radiological fracture healing was 2.8 times shorter in patients with CD34 + cell transplantation than in the historical control group (hazard ratio: 2.81 and 95% confidence interval 1.16–6.85); moreover, no safety concerns were observed.

**Conclusions:**

Our findings strongly suggest that autologous CD34 + cell transplantation is a novel treatment option for fracture nonunion.

**Trial registration:**

UMIN-CTR, UMIN000022814. Registered on 22 June 2016.

**Supplementary Information:**

The online version contains supplementary material available at 10.1186/s12916-023-03088-y.

## Background

A considerable proportion (5–10%) of fractures fail to heal, resulting in delayed union or nonunion [1]. Treatment of fracture nonunion could require multiple operative procedures and prolonged hospitalization, leading to years of disability until attaining union; this has a substantial negative socioeconomic impact [2]. The causes of fracture nonunion are multifactorial, including insufficient mechanical stability and a decline in biological activity [3]. Specifically, compromised blood supply to the fracture site is associated with a high risk of fracture nonunion [4, 5]. Clinically applicable treatment options for enhancing fracture healing are limited; these include bone morphogenetic protein administration [6–22], low-intensity pulsed ultrasound [23], and pulsed electromagnetic fields [23], but the efficacy of these modalities is limited. Therefore, it is essential to develop new treatment options to enhance fracture healing.

Current clinical investigations have focused on cell-based therapies for bone formation as a category of regenerative medicine. CD34 + adult human peripheral blood (PB) cells are candidates for cell-based regenerative therapy, as they contain abundant endothelial progenitor cells (EPCs) and hematopoietic stem cells [24]. Tissue ischemia, along with the secretion of cytokines such as granulocyte colony-stimulating factor (G-CSF), mobilizes EPCs from the bone marrow (BM) into PB; these mobilized EPCs provide sites for nascent neovascularization and differentiate into mature endothelial cells [25, 26]. The therapeutic potential of BM-derived CD34 + cells for neovascularization in limbs and myocardial ischemia has been demonstrated in preclinical and clinical investigations [27–30]. Furthermore, studies have indicated that BM-derived CD34 + cells can differentiate into osteogenic, hematopoietic, and vasculogenic lineages [31–35]. Fractures induce EPC mobilization from the BM into PB, leading to incorporation of the circulating EPCs into the fracture site [36, 37].

We conducted a series of preclinical studies to demonstrate the therapeutic effects of CD34 + cells in fracture healing. First, we showed that the systemic infusion of circulating CD34 + cells from humans into immunodeficient rats with nonhealing fractures contributed to fracture healing by enhancing vasculogenesis and osteogenesis [38]. Next, we attempted local transplantation of CD34 + cells with atelocollagen gel—a bioabsorbable scaffold—in the same animal model and observed a similar effect at a lower dose than that employed for systemic administration [39]. Additionally, we reported the advantages of CD34 + cell transplantation over mononuclear cell transplantation for fracture healing [40]. In this milieu, we performed a phase I/IIa trial involving transplantation of autologous, G-CSF-mobilized CD34 + cells with atelocollagen gel in patients with femoral or tibial fracture nonunion [41]. This early-phase clinical trial, conducted with seven patients, revealed that this cell-based therapy is safe; moreover, the fracture nonunion healed appreciably faster in the study participants than in the historical control patients. There was moderate evidence for clinical application, owing to the limited clinical data available from the seven patients who underwent CD34 + cell transplantation. Subsequently, we conducted a phase III clinical trial involving a larger cohort, and the results are reported herein. We aimed to demonstrate the potential of autologous CD34 + cell transplantation as a novel treatment strategy for fracture nonunion.

## Methods

### Study approval

This phase III clinical trial was designed as a multicenter, single-arm study to evaluate the safety and efficacy of autologous and G-CSF-mobilized CD34 + cells in patients with tibial/femoral fracture nonunion. The study protocol conformed to the Declaration of Helsinki and was approved by the institutional review boards of the seven participating hospitals, which included five university hospitals and two medical centers of the same scale as that of the university hospitals. A clinical trial notification was submitted to the Pharmaceuticals and Medical Devices Agency (PMDA), Japan, and the study was initiated after obtaining the PMDA’s permission. Furthermore, ethical approval was obtained from all seven participating hospitals. All patients provided written informed consent to participate in this trial.

### Study design and criteria for patient enrollment

The following inclusion criteria were used: (a) tibial shaft fracture or femoral fracture (excluding intra-articular fracture); (b) fracture nonunion for over 6 months after previous surgery for fracture treatment; (c) fracture nonunion without infection; (d) plan to undergo autologous bone grafting (ABG) for nonunion surgery; (e) patient age of 20–69 years; and (f) provision of written informed consent. ABG was used when there was a bone gap in the nonunion site or when the type of nonunion was not hypertrophic. The exclusion criteria included a high risk of G-CSF adverse events (AEs), leukapheresis, and lack of CD34 + cells, and are listed in Additional file 1: Table S1 [42].

After obtaining informed consent from patients, the eligibility of each candidate to undergo cell-based therapy was evaluated at each institute. Examinations were undertaken to obtain baseline data. An intradermal reaction test, using atelocollagen gel to detect allergies, was performed; thereafter, the trial was initiated within 4 weeks after registration. The patients were followed for 52 weeks after CD34 + cell transplantation.

The following treatments were prohibited during the trial period: BM and cell-based therapies other than CD34 + cell therapy, gene therapy, or treatment with angiogenesis inducers, such as fibroblast growth factor, study drugs, and medical devices used in other clinical trials, low-intensity pulsed ultrasound, and teriparatide (parathyroid hormone 1–34).

### Setting the primary endpoint

Our previous phase I/II clinical trial revealed that the healing time of fracture nonunion was significantly shorter in patients receiving cell therapy than in historical control group patients [41]. However, the number of patients was small (seven patients), and the study outcomes in both patient groups, that is, patients with femoral and tibial fracture nonunions, were analyzed. Therefore, we decided to set the primary endpoint of the current phase III clinical trial to verify treatment efficacy in a limited population and a higher number of patients. We considered that assessing fracture nonunions of heterogeneous fracture sites would be inappropriate for a comparative study. As such, we focused on tibial shaft fracture nonunions for the primary endpoint assessment. The tibia was selected because it is enveloped by a thinner soft tissue and is estimated to have less vascularity than the femur. Additionally, the involvement of the articular region could affect fracture healing. Hence, we excluded proximal and distal tibial fracture nonunions and only selected tibial shaft fracture nonunions. The tibial shaft is defined as fracture location 42 according to the Arbeitsgemeinschaft für Osteosynthesefragen/Orthopedic Trauma Association classification [43].

### Estimating the required number of patients

We estimated survival curves considering the healing period of fracture nonunion in four patients with tibial fracture nonunions in the previous phase I/II study; furthermore, a historical control group of nine patients with tibial fracture nonunions and a hazard ratio (HR) of 7.41 were estimated. Based on this, considering a 1-year follow-up period and the nine historical control patients, the number of patients required for the rejection of the null hypothesis with an HR of 1, at a two-tailed significance level of 5%, and with a probability of 80% or higher, was calculated to be 15. Additionally, we decided to include up to 10 patients with femoral fracture nonunion as participants in exploratory clinical research during the same study period.

### Historical controls

The historical controls were as follows: (a) patients with uninfected tibial shaft fracture nonunion treated with standard surgeries with ABG before the initiation of the current clinical trial, from November 6, 2007, to April 24, 2012; and (b) patients who were treated by the same group of surgeons and at the same hospital as those in the current trial. The historical control group comprised nine patients (7 men and 2 women) aged 54.9 ± 13.7 (range: 28–73) years. Table 1 summarizes the baseline characteristics of the historical control.

**Table 1:** Characteristics of historical control patients

	Historical control (*n* = 9)
Sex	
Male	7 (78%)
Female	2 (22%)
Age, years	
Mean	54.9
SD	13.7
Min	28
Max	73
Infection	
No	8 (89%)
Previous	1 (11%)
Non-smoker	6 (67%)
DM	0 (0%)
Previous nonunion surgery to treat the nonunion, including ABG	1 (11%)
Exchange of implant in the latest nonunion surgery	
No exchange of plate	1 (11%)
No exchange of IMN	1 (11%)
Plate addition to plate	1 (11%)
Plate addition to IMN	0 (0%)
Plate to plate	1 (11%)
Plate to IMN	0 (0%)
IMN to plate	3 (33%)
IMN to IMN	2 (22%)
IMN to IMN + plate	0 (0%)
EF to plate	0 (0%)
EF to IMN	0 (0%)
EF to IMN + plate	0 (0%)

*ABG* autologous bone grafting, *DM* diabetes mellitus, *EF* external fixator, *IMN* intramedullary nail, *SD* standard deviation

### Mobilization, harvesting, and isolation of CD34 + cells

**Fig. 1:**
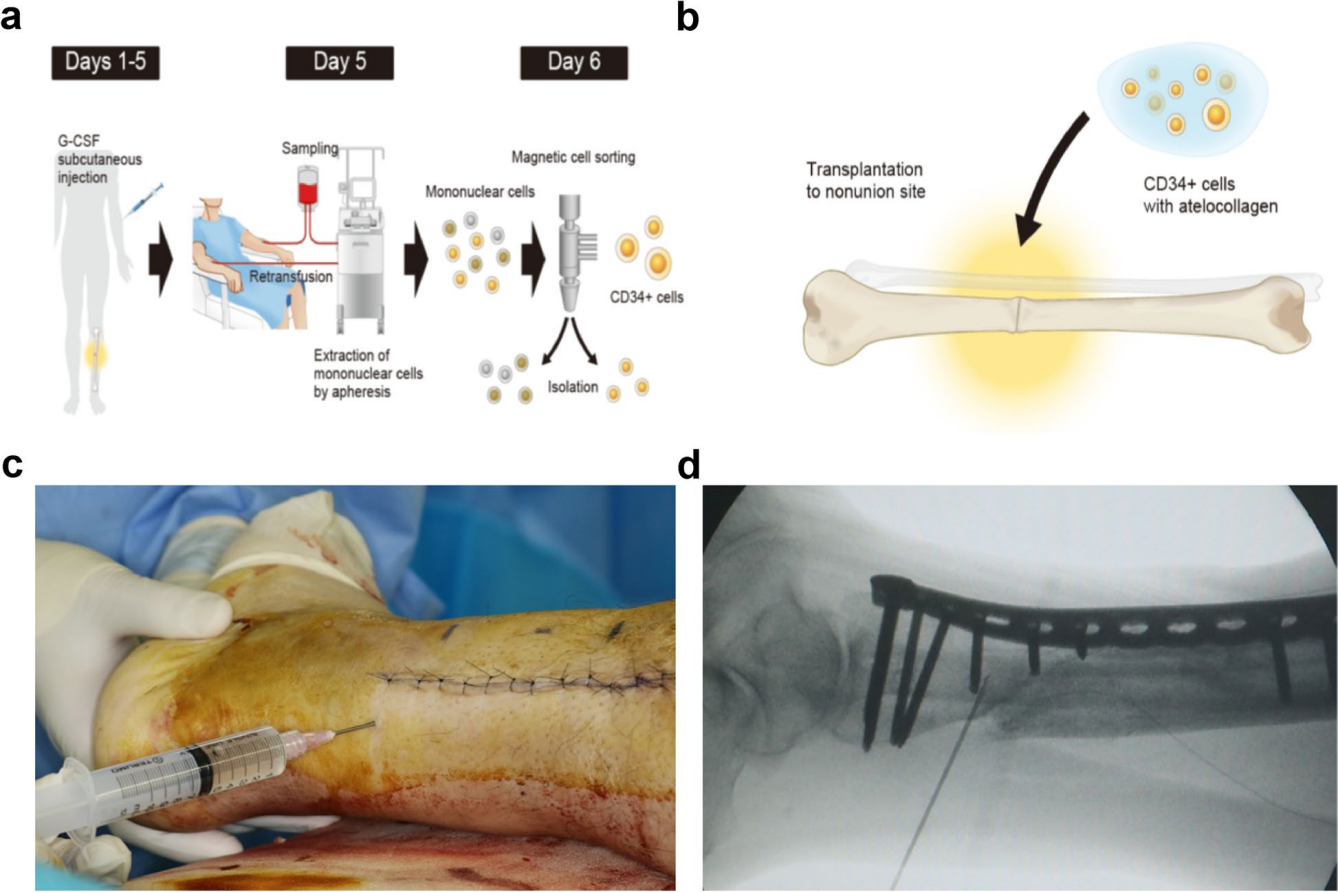
**a** Schematic of the procedure used for autologous CD34 + cell transplantation. **b** Procedure for CD34 + cell transplantation. Following nonunion surgery, local administration (injection) of autologous CD34 + cells dissolved in atelocollagen gel at the nonunion site was performed in a patient with fracture nonunion. **c** Autologous CD34 + cells dissolved in atelocollagen gel were locally injected into the fracture site. **d** Injection was administered under fluoroscopic image control

The scheme of the treatment procedure is shown in Fig. 1a, b. Patients enrolled in this study were subcutaneously administered G-CSF to mobilize CD34 + cells from the BM, containing both EPCs and osteogenic progenitors. The basic dose of G-CSF was 200 μg/m^2^ per day for 5 days. The administration of G-CSF was scheduled to be canceled when the white blood cell count was > 75,000 cells/µL. Leukapheresis was performed to harvest PB mononuclear leukocytes on day 5. The leukapheresis product was kept at a concentration of > 2 × 10^8^ cells/mL in autoplasma until the magnetic separation of CD34 + cells was initiated using the CliniMACS® CD34 Reagent System, CD34 reagent, phosphate-buffered saline/ethylenediaminetetraacetic acid buffer, and a tubing set (Miltenyi Biotec, Bergisch Gladbach, Germany, http://www.miltenyibiotec.com). The time from the completion of CD34 + cell enrichment to delivery to the operation room was 213 ± 67 (range 78–352) min. During this time, tests such as CD34 + cell number, purity, and viability, were conducted, and the surgery room was prepared. All patients received fresh CD34 + cells that were not cryopreserved on the same day of CD34 + cell isolation.

The purity and viability of the isolated CD34 + cells were examined using fluorescence-activated cell sorting (FACS) with the Stem Cell Enumeration Kit (BD Biosciences, Franklin Lakes, NJ, USA) or Stem-Kit (BECKMAN COULTER, Brea, CA, USA) according to the guidelines for CD34 + cell determination using flow cytometry of the Japanese Committee for Clinical Laboratory Standards. The recovery of CD34 + cells was defined using the following formula: recovery of CD34 + cells = (number of CD34 + CD45dim cells after cell isolation) / (number of CD34 + CD45dim cells before cell isolation).

The dose of transplanted cells was the same as that used in the previous phase I/II clinical trial [41]. The standard dose for CD34 + cell transplantation was 5 × 10^5^ cells/kg. The reasons for selecting this dose are as follows. In a previous clinical trial on treating critical limb ischemia, the efficacy and safety were confirmed with the transplantation of up to 1 × 10^6^ cells/kg of CD34 + cells and the use of 10 μg/kg G-CSF [17]. Additionally, in a preclinical study using a rat–human cell xenotransplantation fracture model, the transplantation of 5 × 10^5^ cells/kg of CD34 + cells was more efficacious than that of 5 × 10^4^ cells/kg with acceptable safety [39].

The purity, viability, and recovery rate of the harvested CD34 + cells were assessed using 1 × 10^6^ cells. A CD34 + cell population purity of ≥ 50% and viability of ≥ 70% were set as criteria for cell transplantation. Another group of 1 × 10^6^ harvested CD34 + cells was cryopreserved to investigate the cause of infection after cell transplantation.

If the number of harvested CD34 + cells was ≥ 5 × 10^5^ cells/kg, 5 × 10^5^ cells/kg (standard dose) were transplanted; if it was < 5 × 10^5^ cells/kg, all harvested cells, excluding those for the test and cryopreservation, were transplanted. In this case, if the number of harvested CD34 + cells was ≥ 1 × 10^5^ cells/kg, the efficacy and safety were assessed; if it was < 1 × 10^5^ cells/kg, only safety was assessed.

### Surgery and cell transplantation

Surgery was performed with the participants under general anesthesia. The original plate or intramedullary nail was revised when the mechanical stability of the fracture was insufficient. Autologous cancellous bone was harvested from the iliac crest for grafting. ABG was undertaken following curettage of the fibrous tissue at the nonunion site. After the closure of the surgical wound, autologous CD34 + cell transplantation was performed. Autologous CD34 + cells dissolved in atelocollagen gel (Koken, Tokyo, Japan, http://www.kokenmpc.co.jp/english) were locally injected into the fracture site (Fig. 1c). Atelocollagen gel was used as a bioabsorbable scaffold for the cells. The volume of the gel used was either 4 or 5 mL; surgeons could select either volume, depending on the appropriate amount for each nonunion case. The cells were injected accurately using a syringe at the nonunion site under fluoroscopic imaging (Fig. 1d).

### Rehabilitation protocol

The rehabilitation protocol was not cell therapy specific; a general rehabilitation protocol, applied to patients treated surgically for fractures/fracture nonunion of lower extremities, was followed. Partial weight bearing, range of motion exercise, and muscle training were initiated on postoperative day 1 (next day to the day of surgery). Full weight bearing was allowed 10–12 weeks after surgery.

### Radiological examination of fracture healing

Radiographs of the fracture site were taken at two orthogonal planes (anteroposterior and lateral) before surgery (cell transplantation), immediately after surgery, and at 1, 2, 4, 8, 12, 16, 20, 24, 28, 32, 36, 40, 44, 48, and 52 weeks after the surgery. Computed tomography (CT) scans of the fracture site were also collected before surgery and at 12, 16, 20, 24, 28, 32, 36, 40, 44, 48, and 52 weeks after surgery.

### Definition of fracture healing

Two orthogonal (anteroposterior and lateral) radiographs of each fracture were assessed to determine the amount of callus bridging, absence of fracture lines, and cortical continuity. Radiographic fracture healing was defined as the presence of callus bridging on at least three of the four cortices [44].

### Assessment of fracture healing by an independent data monitoring committee

Radiographs were reviewed, and fracture healing was assessed by five independent reviewers from the clinical trial. All reviewers were expert orthopedic trauma surgeons and were selected as being eligible to judge radiological fracture healing appropriately. They reviewed the radiographs of all patients and historical controls individually and noted whether fractures were healed or not in a dedicated form, wherein the scores of the radiographic union scale in tibial fractures (RUST) [45] and radiographic union score for hip (RUSH) [46] were also noted. The RUST scoring system can help quantitatively assess cortical bone healing and is applied to determining fracture healing of long bones; RUSH can help quantitatively assess the healing of the cortical bone and trabecular bone separately. The completed form was sent to a data management center. For clinical trial patients, CT images obtained at a time point when the reviewer could determine fracture healing were supplemented with radiographs provided to the reviewers. The reviewers confirmed that their assessment using radiography was appropriate. The time point at which three of the five reviewers assessed a fracture to be healed was determined as the time point of fracture healing.

### Assessment of the primary endpoint

The interval between surgery with cell transplantation and radiological fracture healing was assessed as the primary endpoint. Thereafter, this interval was compared between the clinical trial patients and historical controls of tibial shaft fracture nonunion.

### Quality of life and limb function

Health-related quality of life and limb function of the clinical trial patients were assessed before surgery and at 12, 24, 36, and 52 weeks after surgery. Health-related quality of life scores were obtained using the short form-36 version 2 (SF-36v2) questionnaire [47]. We assessed eight-scale scores (vitality, physical functioning, bodily pain, general health perceptions, physical role functioning, emotional role functioning, social role functioning, and mental health), as well as the physical component score (PCS), mental component score (MCS), and role/social component score (RCS). Additionally, limb function was assessed using the Academy of Orthopedic Surgeons (AAOS) Lower Limb Core Scale [48]. This scale includes a normative score (range 0–100), where a value of 0 indicates “worse function,” and a standardized score (range − 15.67 to 56.88), where the mean of a healthy population is 50 [48].

### Safety evaluation

All AEs and medical device failures were recorded. The AEs were coded using the Medical Dictionary for Regulatory Activities and classified according to the system organ class and preferred term, as well as severity. Medical device failure was assessed for the magnetic cell sorter and atelocollagen gel.

Vital signs, including body temperature, blood pressure, and pulse rate, were checked during the screening (baseline) period and at each time point during the clinical trial. Blood examinations, including hematological and biochemical tests, were undertaken during G-CSF administration and leukapheresis; on the day of surgery (cell transplantation); at 1, 4, 7, and 14 days after the surgery; and at 4, 12, 24, and 52 weeks after the surgery. Human anti-mouse antibodies were tested during the screening period and at 24 weeks after surgery [49]. Abnormal changes in the results of blood examination were defined by the evaluation of the physicians-in-charge for this clinical trial, using the assessment standard of the Japanese Society of Chemotherapy [48]. Abdominal ultrasonography was performed during the screening period, especially to detect splenomegaly. Screening for malignancies was undertaken at the baseline and 1 year following transplantation by reviewing CT scans of the chest and abdomen.

### Data management

Data were collected, stored, and managed at a data management center using an electrical data capture system (eClinical Base). Following data input, data cleaning and a logical check were performed to guarantee data quality.

### Statistical analysis

The full analysis set (FAS) included all patients with CD34 + cell transplants with ≥ 1 × 10^5^/ kg while excluding patients deemed ineligible after enrollment. The per-protocol set (PPS) comprised the sub-population, excluding patients who did not complete the treatment protocol. The PPS was used for sensitivity analysis of the primary endpoint. The safety set included all patients who were administered G-CSF.

The treatment efficacy was assessed as follows: The accumulated radiological fracture healing rate after nonunion surgery was estimated using the Kaplan‒Meier method. In patients with tibial fracture nonunion, the accumulated radiological fracture healing rate was compared with that of the historical group using the log-rank test; furthermore, the HR and 95% CI were estimated using a Cox proportional hazard model. The scores of the modified RUST and RUSH at each time point were recorded in the summary statistics. Changes from the baseline were compared using the Wilcoxon signed-rank test. The eight-scale scores and three component scores of SF-36v2 and the scores of the AAOS Lower Limb Core Scale at each time point were also recorded in the summary statistics. Changes from the baseline were compared using the Wilcoxon signed-rank test. For each fracture type, scatter plots and correlation coefficients between the number of transplanted CD34 + cells per body weight and the period from cell transplantation to radiological fracture healing were obtained. The observed AEs were aggregated according to the fracture type, time of occurrence, and cause of occurrence by system organ class and preferred term; thereafter, the frequency and rate of occurrence were calculated. For the measured values considering the blood examination and vital signs, summary statistics from the baseline to each time point were calculated; thereafter, the frequency of abnormal changes in the blood examination results was measured. Missing values were not imputed for primary or secondary endpoints. All analyses were conducted using SAS version 9.4 and R version 4.1.3 (Cary, UC, USA). A *P-*value < 0.05 indicated statistical significance (two-tailed).

## Results

### Patient enrollment

**Fig. 2:**
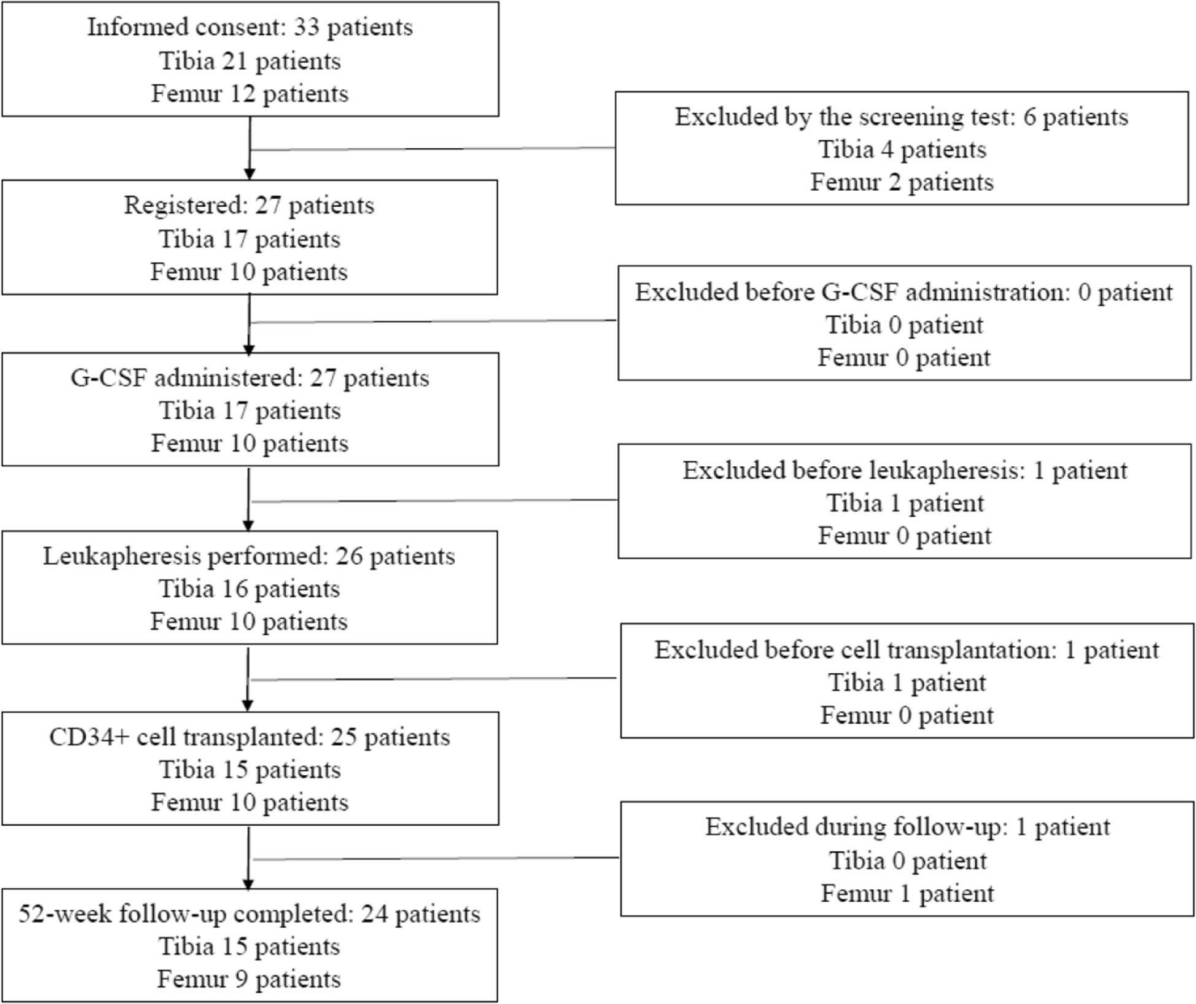
Flow chart of inclusion and exclusion of participants in the clinical trial. Abbreviation: G-CSF, granulocyte colony-stimulating factor

This clinical trial was initiated on June 23, 2016 (the day when informed consent from the first patient was obtained) and was completed on December 24, 2020 (the day when the 52-week follow-up of the last patient was completed). Figure 2 shows a flowchart depicting the inclusion and exclusion of participants in the clinical trial, wherein 27 patients (17 with tibial nonunion and 10 with femoral nonunion) were enrolled. In the FAS, a patient with tibial nonunion was excluded before leukapheresis owing to interstitial pneumonitis, which is an AE associated with G-CSF administration. Another patient with tibial nonunion was also excluded before transplantation, owing to the attainment of a low yield—lower than the lowest limit of the target dose—of CD34 + cells, as determined using FACS. Additionally, a patient with femoral nonunion was excluded from the PPS in the follow-up period after transplantation owing to pregnancy. Therefore, the safety set, FAS, and PPS comprised 17, 15, and 15 patients with tibial nonunion, and 10, 10, and 9 patients with femoral nonunion, respectively.

### Baseline patient characteristics

Table 2 summarizes the baseline characteristics of the current study participants. This was the first time that all patients received CD34 + cell transplantation. Atelocollagen gel (4 mL) was used for all 15 patients with tibial shaft fracture nonunion. Atelocollagen gel (5 mL) was used for nine patients with femur fracture nonunion, whereas a 4-mL dose was used for the remaining patient.

**Table 2:** Characteristics of study patients

	Tibial nonunion (*n* = 15)	Femoral nonunion (*n* = 10)	Total (*n* = 25)
Sex			
Male	11 (73%)	7 (70%)	18 (72%)
Female	4 (27%)	3 (30%)	7 (28%)
Age, years			
Mean	52.7	45.1	49.7
SD	10.1	13.4	11.9
Min	34	22	22
Max	68	66	68
Infection			
No	12 (80%)	9 (90%)	21 (84%)
Previous	3 (20%)	1 (10%)	4 (16%)
Non-smoker	4 (27%)	3 (30%)	7 (28%)
DM	2 (13%)	0 (0%)	2 (8%)
Previous surgery to treat the nonunion, including ABG	1 (7%)	3 (30%)	4 (16%)
Exchange of implants in the current clinical trial			
No exchange of plate	0 (0%)	0 (0%)	0 (0%)
No exchange of IMN	0 (0%)	0 (0%)	0 (0%)
Plate addition to plate	3 (20%)	0 (0%)	3 (12%)
Plate addition to IMN	0 (0%)	2 (20%)	2 (8%)
Plate to plate	0 (0%)	2 (20%)	2 (8%)
Plate to IMN	2 (13%)	1 (10%)	3 (12%)
IMN to plate	1 (7%)	0 (0%)	1 (4%)
IMN to IMN	4 (27%)	3 (30%)	7 (28%)
IMN to IMN + plate	2 (13%)	1 (10%)	3 (12%)
EF to plate	2 (13%)	0 (0%)	2 (8%)
EF to IMN	0 (0%)	1 (10%)	1 (4%)
EF to IMN + plate	1 (7%)	0 (0%)	1 (4%)

*ABG* autologous bone grafting, *DM* diabetes mellitus, *EF* external fixator, *IMN* intramedullary nail, *SD* standard deviation

### Outcome of mobilization, harvesting, and isolation of CD34 + cells

The administration of G-CSF was not terminated in any patient, as the white blood cell count never exceeded 75,000/μL during the administration period. The total dose of G-CSF administered to the FAS group was 1660.6 ± 183.5 μg for patients with tibial nonunion and 1650.6 ± 162.8 μg for patients with femoral nonunion.

The number of cells of the FAS harvested using leukapheresis was 326.63 ± 165.32 (range 0.90 × 10^8^–565.11 × 10^8^) for tibial nonunion and 357.60 ± 227.13 (range 1.95 × 10^8^–628.20 × 10^8^) for femoral nonunion.

The purity of the CD34 + cells of the FAS isolated using CliniMACS was 91.52% ± 4.67% and 91.39% ± 6.13% for patients with tibial and femoral nonunions, respectively. The viability of the FAS CD34 + cells isolated using CliniMACS was 91.84% ± 5.38% and 91.96% ± 4.92% for patients with tibial and femoral nonunions, respectively (Table 3). One hundred percent of the FAS with the CD34 + cell product met the release criteria (purity ≥ 50% and viability ≥ 70%).

**Table 3:** Purity, viability, and recovery of the transplanted autologous CD34 + cells

		Femoral nonunion (*n* = 10)	Tibial nonunion (*n* = 15)	Total patients (*n* = 25)
Purity (%)	Mean ± SD	91.4 ± 6.1	91.5 ± 4.7	91.5 ± 5.2
	Min.–Max	77.0–97.1	83.3–98.3	77.0–98.3
	Median (IQR)	92.5 (89.6–96.1)	91.4 (87.5–95.5)	91.6 (89.4–95.6)
Viability (%)	Mean ± SD	92.0 ± 4.9	91.8 ± 5.4	91.9 ± 5.1
	Min.–Max	83.0–98.0	78.3–97.1	78.3–98.0
	Median (IQR)	93.1 (88.7–95.6)	93.3 (91.2–95.7)	93.3 (91.1–95.6)
Recovery (%)	Mean ± SD	65.5 ± 17.7	70.8 ± 8.9	68.7 ± 13.1
	Min.–Max	31.8–93.7	55.5–91.6	31.8–93.7
	Median (IQR)	64.0 (56.2–78.4)	70.4 (65.1–76.8)	70.2 (60.7–76.8)

*IQR* interquartile range, *SD* standard deviation

The number of transplanted CD34 + cells of the FAS was 28.82 ± 6.80 (× 10^6^) and 26.27 ± 7.13 (× 10^6^) for the tibial and femoral nonunion patients, respectively. A full dose of CD34 + cells (5 × 10^5^ cells/kg) was transplanted into 73% of the 15 FAS patients exhibiting tibial nonunion and 70% of the 10 FAS patients with femoral nonunion. The number of transplanted CD34 + cells lower than the full dose was 3.6 × 10^5^, 4.1 × 10^5^, 4.4 × 10^5^, and 4.9 × 10^5^ cells/kg for four patients with tibial nonunion and 1.3 × 10^5^, 3.7 × 10^5^, and 4.0 × 10^5^ cells/kg for three patients with femoral nonunion. None of the patients received fewer than 1 × 10^5^ cells/kg of CD34 + cells. The number of transplanted CD34 + cells in each patient is shown in Additional file 1: Table S2.

Cell transplantation was terminated in one patient because the yield of CD34 + cells obtained using FACS was lower than the lowest limit of the target dose. However, post-procedural verification revealed that the cell number was underestimated because the gate setting in FACS was incorrect. Verification revealed that the cell number met the release criteria.

### Primary outcomes

#### Healing period in patients with tibial nonunion

**Fig. 3:**
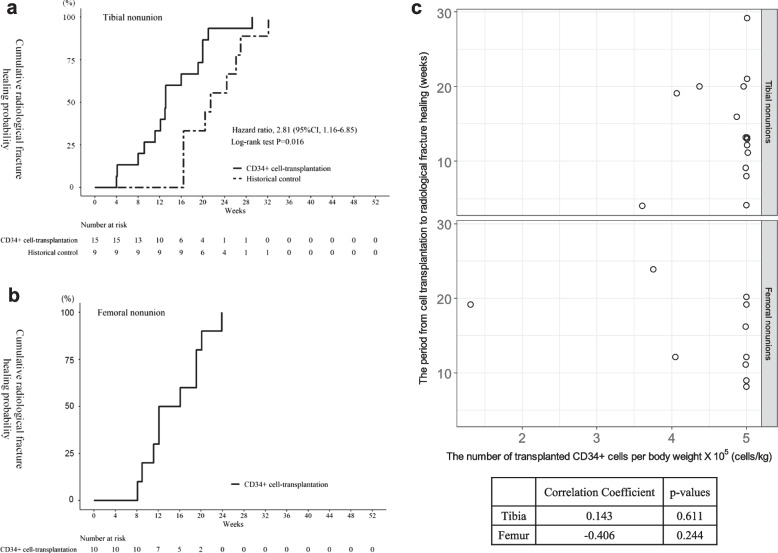
Healing of fracture nonunions of clinical trial patients subjected to CD34 + cell transplantation. Kaplan–Meier curve for the healing of fracture nonunions. The vertical and horizontal axes depict the cumulative radiological fracture healing probability and the weeks after the nonunion surgery, respectively. The solid line represents trial patients subjected to CD34 + cell transplantation, and the dotted line represents the historical controls. **a** Tibial nonunions, comparing the clinical trial patients subjected to CD34 + cell transplantation and the historical controls. **b** Femoral nonunions. **c** Scatter plots and correlation coefficients between the number of transplanted CD34 + cells per body weight and the period from cell transplantation to radiological fracture healing. Upper: tibial nonunions, and lower: femoral nonunions

All nonunions healed and showed bone union. The primary outcome, the period from cell transplantation to radiological fracture healing, was significantly shorter in the CD34 + cell transplantation group than in the historical control group for tibial nonunion (*P* = 0.016, log-rank test) (Fig. 3a). The HR of the accumulated rate of radiological fracture healing in the trial patients compared to the historical control was 2.81 (95% CI 1.16–6.85). The cumulative rate of radiological fracture healing was 6.7% at 4 weeks, 20.0% at 8 weeks, 33.3% at 12 weeks, and 66.7% at 16 weeks in patients with tibial nonunion who received CD34 + cells, whereas no complete healing was observed by week 16 in the historical control. The period from nonunion surgery to radiological fracture healing was 99.5 ± 47.7 days in patients who received CD34 + cells and 156.2 ± 38.8 days in the historical controls. The healing periods of patients with full or lower doses of CD34 + cells are presented in Additional file 1: Tables S2 and S3.

#### Healing period in patients with femoral nonunion

All nonunions healed and showed bone union. The cumulative rate of radiological fracture healing was 30.0% at 12 weeks and 50.0% at 16 weeks (Fig. 3b). The periods from cell transplantation to radiological fracture healing in all patients with femoral nonunion and in patients transplanted full or lower doses of CD34 + cells are shown in Additional file 1: Tables S2 and S3.

#### Correlation of cell dose with efficacy

No significant correlation was observed between the radiological healing period and the number of transplanted CD34 + cells in either tibial or femoral nonunion patients (Fig. 3c).

### Secondary outcomes

#### Modified radiographic union scale in tibial fractures and radiographic union score for hip

**Fig. 4:**
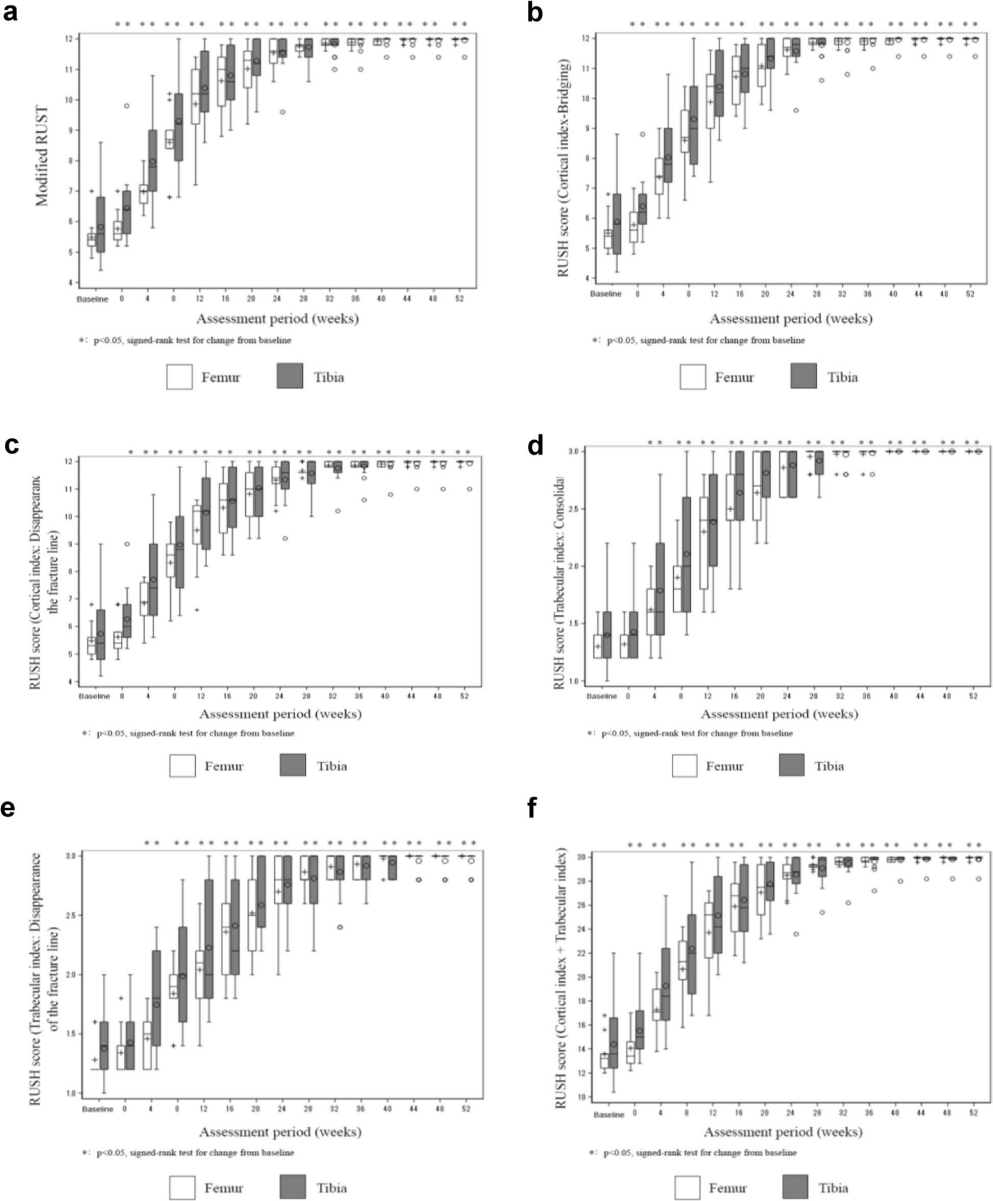
RUST and RUSH scores of clinical trial patients subjected to CD34 + cell transplantation. **a** The vertical axis indicates the RUST score, and the horizontal axis represents the assessment period of 52 weeks. The white boxes represent patients with femoral fracture nonunion, and the gray boxes represent patients with tibial shaft fracture nonunion. For the RUSH score, the vertical axes represent **b** cortical index-bridging, **c** cortical index-disappearance of the fracture line, **d** trabecular index-consolidation, **e** trabecular index-disappearance of the fracture line, and **f** cortical index plus trabecular index. Error bars represent standard deviation. Abbreviations: RUSH, radiographic union score for hip; RUST, radiographic union scale in tibial fractures

The modified RUST (Fig. 4a) and RUSH (Fig. 4b–f) scores increased with time after CD34 + cell therapy in patients with both tibial and femoral nonunions. The differences in the scores from the baseline (scores before transplantation) were substantially higher at each time point after transplantation for modified RUST and cortical index-bridging, cortical index-disappearance of the fracture line, trabecular index-consolidation, and trabecular index-disappearance of the fracture line of RUSH for both patients with tibial and femoral nonunions. Regarding the overall assessment (healed or not healed) section of the RUSH, the number of healed patients exceeded that of unhealed patients with statistical significance for the first time at 12 and 16 weeks for patients with tibial and femoral nonunions, respectively.

#### SF-36

**Fig. 5:**
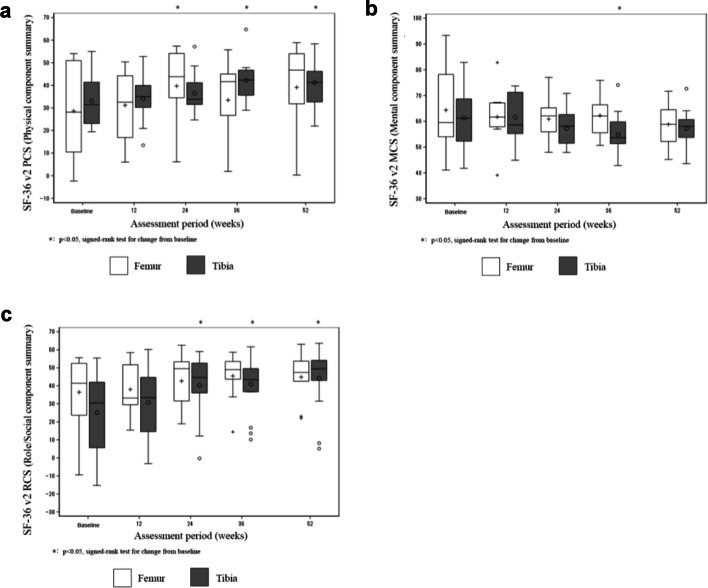
SF-36v2 component summary score of clinical trial patients subjected to CD34 + cell transplantation. The patients were followed up for 52 weeks, with SF-36v2 summary scores assessed at the indicated time points. The vertical axis depicts the **a** PCS, **b** MCS, and **c** RCS. The white boxes represent patients with femoral fracture nonunion, and the gray boxes represent patients with tibial shaft fracture nonunion. Error bars represent standard deviation. Abbreviations: MCS, mental component score; PCS, physical component score; RCS, role/social component score; SF-36v2, short form survey-36 version 2

The SF-36v2 PCS was substantially higher at 36 and 52 weeks after transplantation than at baseline for patients with tibial nonunion and substantially higher at 24 weeks after transplantation compared to the baseline value in patients with femoral nonunion (Fig. 5a). The MCS of SF-36v2 was substantially lower at 36 weeks after transplantation compared to the baseline value in patients with tibial nonunion; moreover, no significant difference in the MCS at any time point compared to the baseline value in patients with femoral nonunion (Fig. 5b). The RCS of SF-36v2 was substantially higher at 24, 36, and 52 weeks after transplantation compared to the baseline value in patients with tibial nonunion, and there was no significant difference in the RCS at any time point compared to the baseline value in patients with femoral nonunion (Fig. 5c). The eight-scale scores of SF-36v2 are shown in Additional file 2: Figure S1.

#### AAOS lower limb core scale

**Fig. 6:**
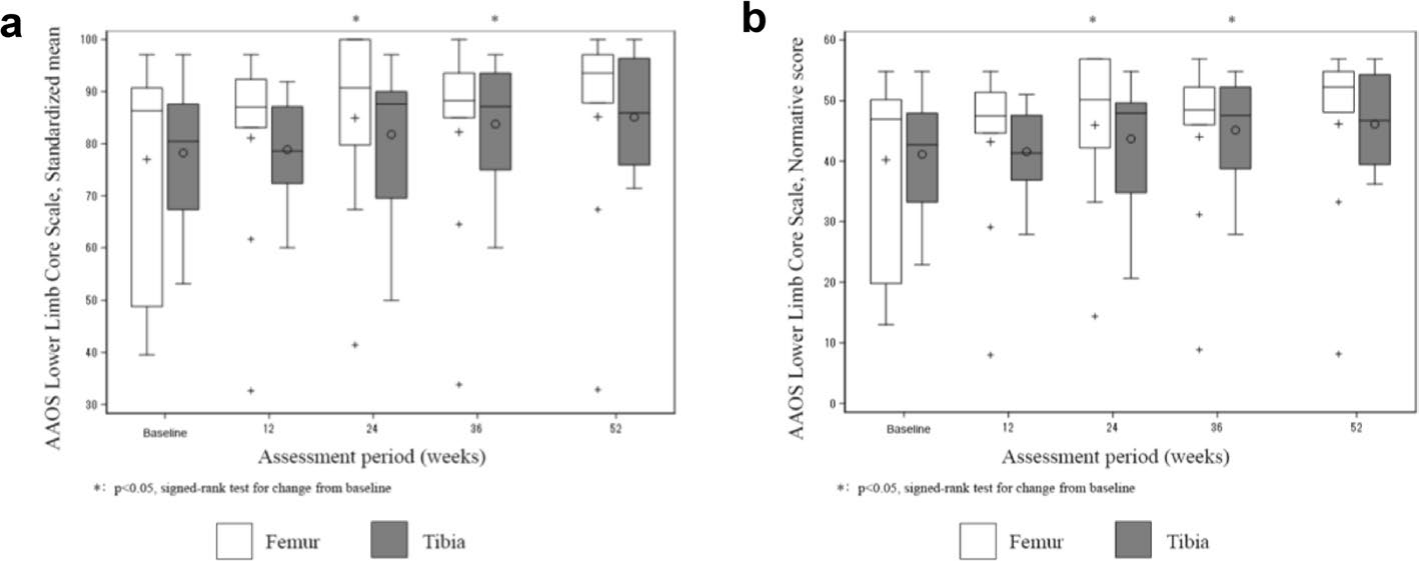
AAOS Lower Limb Core Scale of clinical trial patients subjected to CD34 + cell transplantation. The patients were followed up for 52 weeks, with AAOS outcome scores evaluated at the indicated time points. The vertical axis depicts the **a** standardized mean and **b** normative score. The white boxes represent patients with femoral fracture nonunion, and the gray boxes represent patients with tibial shaft fracture nonunion. Error bars represent standard deviation. AAOS, American Academy of Orthopedic Surgeons

This scale revealed an improvement in the clinical outcomes in patients with tibial and femoral nonunions (Fig. 6a, b). The standardized mean and normative scores were substantially higher at 36 weeks after transplantation in patients with tibial nonunion and at 24 weeks in patients with femoral nonunion (than the baseline values).

### Safety evaluation

The AEs related to G-CSF administration were recorded in 14 (82.4%) patients with tibial nonunion and 10 (100%) patients with femoral nonunion among the 27 patients who were administered G-CSF (Table 4). The AEs related to leukapheresis were recorded in 9 (56.3%) patients with tibial nonunion and 7 (70%) patients with femoral nonunion among the 26 patients who underwent leukapheresis (Table 4). However, no severe AEs or deaths were observed in patients who received G-CSF or those who underwent leukapheresis, except for one patient who had a G-CSF-induced serious AE, interstitial pneumonitis, leading to treatment discontinuation before leukapheresis. Additional file 1: Tables S4 and S5 list AEs related to G-CSF administration and leukapheresis, which occurred in three or more patients. AEs were recorded in 15 patients with tibial nonunion and 10 patients with femoral nonunion who received CD34 + cell transplantation during the study period (Table 4). All AEs were mild-to-moderate, transient, and disappeared without any permanent damage. No severe AEs were observed in either case. There were no deaths or AEs associated with the termination of transplantation during the study. Moreover, no AEs related to the CliniMACS system, atelocollagen gel (medical devices), or medical device failures were observed in patients who received CD34 + cell transplantation. The AEs that occurred in three or more patients are listed in Additional file 1: Tables S4–S6.

**Table 4:** Summary of AEs

	Tibial nonunion	Femoral nonunion	Total patients
	Number of patients (%)	Number of patients (%)	Number of patients (%)
**Patients receiving G-CSF**	(*n* = 17)	(*n* = 10)	(*n* = 27)
AEs related to G-CSF	14 (82)	10 (100)	24 (89)
**Patients undergoing leukapheresis**	(*n* = 16)	(*n* = 10)	(*n* = 26)
AEs related to apheresis	9 (56)	7 (70)	16 (62)
**Patients receiving CD34 + cells**	(*n* = 15)	(*n* = 10)	(*n* = 25)
AEs	15 (100)	10 (100)	25 (100)
AEs related to medical devices	0 (0)	0 (0)	0 (0)
Magnetic cell sorter	0 (0)	0 (0)	0 (0)
Atelocollagen gel	0 (0)	0 (0)	0 (0)
AEs unrelated to medical devices	15 (100)	10 (100)	25 (100)
CD34 + cells	0 (0)	0 (0)	0 (0)
Cell transplantation	0 (0)	0 (0)	0 (0)
Nonunion surgery	14 (93)	10 (100)	24 (96)
Comorbidity	0 (0)	0 (0)	0 (0)
Others	15 (100)	10 (100)	25 (100)
Severe AEs	0 (0)	0 (0)	0 (0)
Serious AEs	2 (13)	2 (20)	4 (16)
Death	0 (0)	0 (0)	0 (0)
AEs to stop cell transplantation	0 (0)	0 (0)	0 (0)
Medical device failure	0 (0)	0 (0)	0 (0)

*AE* adverse event, *G-CSF* granulocyte colony-stimulating factor

In addition, severe AEs were observed in 2 patients with tibial nonunion and 2 patients with femoral nonunion from the first day of G-CSF administration to the last day of follow-up (Table 4). Among the patients with tibial nonunion who experienced severe AEs, one experienced an anorectal abscess, and the other had a postoperative surgical site infection. Among the patients with femoral nonunion who experienced severe AEs, one had a fever, and the other had loosening of a screw used for bone fixation, which was considered nonunion surgery-related. The loosening of the screw did not affect bone healing, and all other severe AEs were fully resolved during the study period.

Human anti-mouse antibodies were detected in only one patient during the screening period. This patient received fewer CD34 + cells than the target dose; hence, cell transplantation was terminated, and the antibody test was not performed 24 weeks after surgery. The human anti-mouse antibody was not identified during the screening period nor 24 weeks after the surgery in the remaining patients. In the clinical laboratory evaluation, abnormal changes, which were observed in ≥ 30% of all patients, included a decrease in the red blood cell count, hemoglobin level, and hematocrit, and an increase in creatinine phosphokinase levels, which were observed after cell therapy/nonunion surgery. These abnormal findings were common after surgery.

## Discussion

This clinical trial revealed the efficacy and safety of CD34 + cell transplantation in patients with fracture nonunion. Harvesting, isolation, and transplantation of CD34 + cells were performed safely in all patients. No malignant tumors were identified during the study period. Mild-to-moderate events related to G-CSF administration and leukapheresis were frequent but transient. These outcomes indicated the feasibility and overall safety of CD34 + cell therapy in patients with fracture nonunion. In terms of efficacy, the results of this trial revealed that fracture nonunions heal fully and more rapidly with our novel regeneration therapy using autologous CD34 + cells than with the pre-existing standard treatment.

Surgical intervention is the primary option for fracture nonunion treatment, and ABG is often performed. However, a certain proportion of patients do not achieve bone union even after ABG [6–13]. Recently, several alternative therapies that supplement biological activity have been used to treat fracture nonunions, such as bone morphogenetic protein [6–22], BM aspirate [50–52], BM-derived mesenchymal stem cell (MSC) [53–56], and adipose-derived stem cell administration [57]. A healing rate of 100% has not been achieved in most clinical investigations, and the 100% healing rate in the current clinical trial may therefore be considered outstanding. MSCs can differentiate into osteoblasts; therefore, MSCs are applied to cell-based bone regeneration therapy with the expectation of replacing the defective or missing osteoblastic activity for fracture nonunion patients. In contrast, CD34 + cells can differentiate into osteogenic, hematopoietic, and vasculogenic lineages. Therefore, CD34 + cells can contribute to accelerating fracture nonunion healing by enhancing osteogenesis and vasculogenesis. The “diamond concept” summarizes the necessary factors to heal bone successfully [58]. The “diamond concept” asserts that vascularity is an important factor in addition to osteogenic activity to heal bone successfully. From this perspective, CD34 + cells are more useful than MSCs in accelerating fracture nonunion healing. In addition, using MSCs need to be expanded in cell culture. Our method using CD34 + cells does not require culture expansion, minimizing the risk of infection or contamination, and eliminating the waiting period associated with culture expansion before cell transplantation. We believe these are key advantages of our cell therapy strategy using CD34 + cells. BM aspiration is an invasive procedure involving the harvest of BM or BM-derived mesenchymal stem cells. Here, we harvested CD34 + cells through leukapheresis, which is less invasive than BM aspiration; we believe this to be a distinct advantage of our cell therapy method. Collectively, the results of this phase III clinical trial strongly support that autologous CD34 + cell transplantation could be a novel option for fracture nonunion treatment.

This study had certain limitations. Although a randomized controlled trial is ideal to confirm the efficacy and safety of this cell therapy, we selected a nonrandomized, single-arm study. The principal reason for our selection was ethical issues with establishing placebo controls for patients with fracture nonunion, as a long-standing condition would interrupt daily activities. Additionally, the clinical background of the patients with fracture nonunion varied widely. Although we standardized the fracture location and limited this to the diaphysis of the tibia for comparison between the trial patients and historical controls, other factors, including the severity of the initial trauma, soft tissue condition, and duration of the nonunion, could not be standardized. Additionally, the heterogeneity of the fracture fixation devices used to fix the initial fracture and nonunion could be considered to have affected the healing time of the nonunion. Furthermore, the number of participants was small, and the study focused only on the tibia and femur.

The results of the current clinical trial suggest the potential efficacy of CD34 + cell therapy for nonunion; however, here, ABG with CD34 + cell therapy was administered to all patients. Consequently, we could not evaluate the efficacy of CD34 + cell therapy alone for healing fracture nonunions. Nevertheless, we used a historical control group of patients treated with ABG for comparison; therefore, the differences in the treatment methods reflect the CD34 + cell-based therapy, and the result that the treated fracture nonunions healed faster than the control injuries could be attributed to the CD34 + cell therapy.

In this cell therapy, G-CSF was systemically administered to patients to mobilize CD34 + cells from the BM to the PB. Animal studies have shown that G-CSF contributes to bone repair [59–61]. Therefore, we cannot exclude the possibility that G-CSF contributed to treatment efficacy in the current clinical trial. The administration of G-CSF is essential to harvest enough cells to predict the treatment outcomes; moreover, excluding G-CSF from this therapy was not feasible. To elucidate the safety and efficacy of CD34 + cell therapy alone, randomized clinical trials with an appropriate control group receiving either G-CSF or placebo are warranted.

Less than the full dose of CD34 + cells was administered to 4 patients with tibial nonunion and 3 patients with femoral nonunion. All these patients achieved bone union, suggesting no differences between patients given the full or reduced dose of CD34 + cells in terms of the primary outcome. In addition, no significant correlation was detected between the radiological healing period and the number of transplanted CD34 + cells in either tibial or femoral nonunion patients. However, the interval between the nonunion surgery and radiological healing differed among these patients. We speculate that this could be attributed to the small sample size and the relatively minor variation in cell dose in this study. Unfortunately, the number of patients was insufficient to statistically compare the effects between the full and lower doses. Hence, future studies should be conducted with a larger sample size of patients.

## Conclusions

The results of this phase III clinical trial strongly suggest that autologous CD34 + cell transplantation could be a novel treatment option for fracture nonunion.

### Supplementary Information

**Additional file 1: Table S1.** Exclusion criteria for this clinical trial. **Table S2.** Number of transplanted CD34+ cells and the interval between the nonunion surgery and radiological healing in each patient. **Table S3.** Interval (days) between nonunion surgery and radiological healing. **Table S4. **AEs related to G-CSF among patients administered G-CSF. **Table S5.** AEs related to leukapheresis among patients who underwent leukapheresis. **Table S6.** AEs and medical device failures in patients receiving CD34+ cell transplantation.

**Additional file 2: Figure S1.** SF-36v2 scores of clinical trial patients treated with CD34+ cell transplantation assessed at different time-points. The horizontal axis shows the assessment period of 52 weeks. The vertical axis represents the SF-36v2 scores for a) physical functioning, b) physical role, c) bodily pain, d) general health, e) vitality, f) social functioning, g) emotional role, and h) mental health. The white boxes represent patients with femoral fracture nonunion, and gray boxes represent patients with tibial shaft fracture nonunion. Error bars represent standard deviation.

## Data Availability

All data supporting the findings of this study are available within the main text or the additional files. Data sharing will be considered on reasonable request to the corresponding author.

## Abbreviations

AAOS: American Academy of Orthopedic Surgeons
ABG: Autologous bone grafting
AE: Adverse event
BM: Bone marrow
CI: Confidence interval
EPC: Endothelial progenitor cell
FACS: Fluorescence-activated cell sorting
FAS: Full analysis set
G-CSF: Granulocyte colony-stimulating factor
HR: Hazard ratio
PB: Peripheral blood
PPS: Per-protocol set
RUSH: Radiographic union score for hip
RUST: Radiographic union scale in tibial fractures
SF-36v2: Short form-36 questionnaire version 2
