# Supplementary material for: Phase III clinical trial of autologous CD34 + cell transplantation to accelerate fracture nonunion repair
Source: BMC Med. 2023 Oct 5;21:386. doi: 10.1186/s12916-023-03088-y (PMC10557317; doi:10.1186/s12916-023-03088-y)
Supplement: Supplementary file 1 — Additional file 1: Table S1. Exclusion criteria for this clinical trial. Table S2. Number of transplanted CD34+ cells and the interval between the nonunion surgery and radiological healing in each patient. Table S3. Interval (days) between nonunion surgery and radiological healing. Table S4. AEs related to G-CSF among patients administered G-CSF. Table S5. AEs related to leukapheresis among patients who underwent leukapheresis. Table S6. AEs and medical device failures in patients receiving CD34+ cell transplantation. [file 12916_2023_3088_MOESM1_ESM.docx]

**Table S1.** Exclusion criteria for this clinical trial

1. Patients with congenital pseudoarthrosis.
2. Patients with multiple nonunions.
3. Patients receiving radiation therapy or chemotherapy to treat cancer; and steroids or immunosuppressive drugs.
4. Patients with lower-extremity paralysis caused by neurological disorders.
5. Patients with allergies or hypersensitivity to human serum albumin.
6. Patients with allergies or hypersensitivity to calf-derived proteins.
7. Patients with a history of anaphylaxis.
8. Patients with a history of hypersensitivity or severe side effects to G-CSF and collagen products.
9. Patients with a history of hypersensitivity or severe side effects to mouse-derived proteins, iron, or iron dextran.
10. Patients not allowed to stop anticoagulant or antiplatelet drugs perioperatively.
11. Patients with cancer or a history of cancer within the last 5 years.
12. Patients with proliferative diabetic retinopathy (BII to BV according to the new Fukuda classification*).
13. Patients with unstable ischemic heart disease, myocardial infarction, brain infarction, intracerebral bleeding, or transient ischemic attack within the last three months.
14. Patients with myeloproliferative disorders or myelodysplastic syndromes.
15. Patients with severe liver, kidney, heart, lung, blood, and endocrine diseases.
16. Patients exhibiting positive results considering the intradermal reaction test to atelocollagen gel (4-week observation required).
17. Patients with a history or family history of autoimmune diseases, such as rheumatoid arthritis, psoriatic arthritis, systemic lupus erythematosus, dermatomyositis, polymyositis, Hashimoto thyroiditis, Graves’ disease, polyarteritis, scleroderma, ulcerous colitis, Crohn’s disease, Sjögren’s syndrome, Reiter’s syndrome, and mixed connective tissue disease.
18. Patients with interstitial pneumonia or a history of interstitial pneumonia.
19. Patients with white blood cell count < 4000/μL or > 10000/μL.
20. Patients with platelet count < 100000/μL.
21. Patients with a hemoglobin level < 8 g/dL.
22. Patients with pathological splenomegaly, as indicated by abdominal ultrasonography and computed tomography.
23. Patients who underwent surgery for the treatment of nonunions within the last 6 months.
24. Patients who planned to undergo any surgery within the clinical trial period.
25. Patients with positive results with respect to HBs antigen, HCV antibodies, HIV antibodies, HTLV antibodies, and serological tests for syphilis (patients positive for HCV antibodies and no HCV-RNA were not excluded).
26. Patients participating in other clinical trials within 3 months of participation in this clinical trial.
27. Female patients currently pregnant or those planning pregnancy, or patients who could not undertake anticonception.
28. Patients with alcohol abuse or drug dependence.
29. Patients judged to be ineligible for inclusion in this clinical trial by the principal investigator or sub-investigator.

G-CSF: granulocyte colony-stimulating factor, HB: hepatitis B, HCV: hepatitis C, HIV: human immunodeficiency virus, HTLV: human T-cell leukemia virus, RNA: ribonucleic acid

**Table S2.** Number of transplanted CD34+ cells and the interval between the nonunion surgery and radiological healing in each patient

| Case number | Fracture site | Transplanted CD34+ cells（×10^6^ cells） | Weight （kg） | Transplanted CD34+ cells（×10^5^ cells/kg） | Interval between nonunion surgery and radiological healing  (d) |
| --- | --- | --- | --- | --- | --- |
| 001-001 | tibia | 22.4 | 62.3 | 3.6 | 28 |
| 003-005 | tibia | 25.6 | 51.1 | 5.0 | 92 |
| 003-006 | tibia | 23.5 | 57.6 | 4.1 | 134 |
| 003-007 | tibia | 31.2 | 62.4 | 5.0 | 92 |
| 003-008 | tibia | 25.6 | 51.1 | 5.0 | 147 |
| 003-009 | tibia | 24.9 | 49.7 | 5.0 | 204 |
| 003-010 | tibia | 34.2 | 68.4 | 5.0 | 64 |
| 003-015 | tibia | 25.8 | 59.0 | 4.4 | 140 |
| 003-016 | tibia | 45.6 | 91.1 | 5.0 | 56 |
| 003-017 | tibia | 21.1 | 42.1 | 5.0 | 78 |
| 003-018 | tibia | 35.8 | 71.5 | 5.0 | 91 |
| 003-019 | tibia | 25.8 | 51.5 | 5.0 | 85 |
| 006-001 | tibia | 25.3 | 50.6 | 5.0 | 29 |
| 007-001 | tibia | 27.5 | 55.3 | 5.0 | 140 |
| 007-002 | tibia | 38.0 | 77.9 | 4.9 | 112 |
| 002-001 | femur | 32.0 | 64.0 | 5.0 | 85 |
| 003-001 | femur | 28.0 | 56.0 | 5.0 | 78 |
| 003-002 | femur | 24.2 | 48.4 | 5.0 | 134 |
| 003-004 | femur | 9.0 | 68.7 | 1.3 | 134 |
| 003-011 | femur | 22.2 | 44.4 | 5.0 | 113 |
| 003-012 | femur | 30.7 | 61.3 | 5.0 | 141 |
| 003-014 | femur | 33.2 | 66.4 | 5.0 | 57 |
| 005-001 | femur | 23.5 | 62.7 | 3.7 | 167 |
| 006-002 | femur | 29.5 | 59.0 | 5.0 | 63 |
| 006-003 | femur | 30.4 | 75.1 | 4.0 | 85 |

**Table S3.** Interval (days) between nonunion surgery and radiological healing

|  |  | Total | Full dose of CD34+ cells (5×10^5^ cells/kg) | Lower than the full dose |
| --- | --- | --- | --- | --- |
| Tibial nonunion | Mean ± SD | 99.5 ± 47.7 | 98.0 ± 48.8 | 103.5 ± 51.8 |
|  | Min.–Max. | 28–204 | 29–204 | 28–140 |
|  | Median (IQR) | 92 (64–140) | 91 (64–140) | 123 (70–137) |
| Femoral nonunion | Mean ± SD | 105.7 ± 37.2 | 95.9 ± 33.7 | 128.7 ± 41.3 |
|  | Min.–Max. | 57–167 | 57–141 | 85–167 |
|  | Median (IQR) | 99 (78–134) | 85 (63–134) | 134 (85–167) |

IQR: interquartile range; SD: standard deviation

**Table S4.** AEs related to G-CSF among patients administered G-CSF

| SOC / PT | Tibial nonunion  (n = 17) | Femoral nonunion  (n = 10) | Total patients  (n = 27) |
| --- | --- | --- | --- |
|  | Number of patients (%) | Number of patients (%) | Number of patients (%) |
| Number of patients with AEs | 14 (82) | 10 (100) | 24 (89) |
| General disorders and administration site conditions | 0 (0) | 3 (30) | 3 (11) |
| Investigations | 14 (82) | 7 (70) | 21 (78) |
| Blood alkaline phosphatase increased | 13 (77) | 7 (70) | 20 (74) |
| Blood lactate dehydrogenase increased | 12 (71) | 6 (60) | 18 (67) |
| C-reactive protein increased | 7 (41) | 3 (30) | 10 (37) |
| Platelet count decreased | 2 (12) | 1 (10) | 3 (11) |
| Metabolism and nutrition disorders | 2 (12) | 1 (10) | 3 (11) |
| Hyperuricemia | 2 (12) | 1 (10) | 3 (11) |
| Musculoskeletal and connective tissue disorders | 10 (59) | 7 (70) | 17 (63) |
| Back pain | 8 (47) | 7 (70) | 15 (56) |
|  | | | |
| MedDRA Version 23.0 | | | |
| Preferred terms of adverse events that occurred in more than or equal to three patients. | | | |

AE: adverse events; G-CSF: granulocyte colony-stimulating factor; PT: preferred term; SOC: system organ class

**Table S5.** AEs related to leukapheresis among patients who underwent leukapheresis

| SOC / PT | Tibial nonunion  (n = 16) | Femoral nonunion  (n = 10) | Total patients  (n = 26) |
| --- | --- | --- | --- |
|  | Number of patients (%) | Number of patients (%) | Number of patients  (%) |
| Number of patients with AEs | 9 (56) | 7 (70) | 16 (62) |
| Injury, poisoning, and procedural complications | 5 (31) | 1 (10) | 6 (23) |
| Citrate toxicity | 5 (31) | 1 (10) | 6 (23) |
| Two investigations | 6 (38) | 5 (50) | 11 (42) |
| Platelet count decreased | 5 (31) | 4 (40) | 9 (35) |
| Nervous system disorders | 1 (6) | 2 (20) | 3 (12) |
| Hypoesthesia | 1 (6) | 2 (20) | 3 (12) |
|  | | | |
| MedDRA Version 23.0 | | | |
| Preferred terms of adverse events that occurred in more than or equal to three patients. | | | |

AE: adverse events; PT: preferred term; SOC: system organ class

**Table S6.** AEs and medical device failures in patients receiving CD34+ cell transplantation

| SOC / PT | Tibial nonunion  (n = 15) | Femoral nonunion  (n = 10) | Total patients  (n = 25) |
| --- | --- | --- | --- |
|  | Number of patients (%) | Number of patients (%) | Number of patients  (%) |
| Number of patients with AEs | 15 (100) | 10 (100) | 25 (100) |
| Gastrointestinal disorders | 8 (53) | 8 (80) | 16 (64) |
| Constipation | 3 (20) | 3 (30) | 6 (24) |
| Nausea | 2 (13) | 4 (40) | 6 (24) |
| Vomiting | 3 (20) | 2 (20) | 5 (20) |
| General disorders and administration site conditions | 3 (20) | 4 (40) | 7 (28) |
| Pyrexia | 2 (13) | 2 (20) | 4 (16) |
| Infections and infestations | 8 (53) | 8 (80) | 16 (64) |
| Nasopharyngitis | 4 (27) | 5 (50) | 9 (36) |
| Injury, poisoning, and procedural complications | 14 (93) | 10 (100) | 24 (96) |
| Procedural pain | 12 (80) | 8 (80) | 20 (80) |
| Anemia postoperative | 11 (73) | 8 (80) | 19 (76) |
| Wound complication | 2 (13) | 2 (20) | 4 (16) |
| Investigations | 11 (73) | 8 (80) | 19 (76) |
| C-reactive protein increased | 6 (40) | 2 (20) | 8 (32) |
| Blood creatine phosphokinase increased | 4 (27) | 3 (30) | 7 (28) |
| Platelet count decreased | 4 (27) | 2 (20) | 6 (24) |
| White blood cell count decreased | 1 (7) | 2 (20) | 3 (12) |
| Metabolism and nutrition disorders | 10 (67) | 5 (50) | 15 (60) |
| Hypercalcemia | 10 (67) | 2 (20) | 12 (48) |
| Musculoskeletal and connective tissue disorders | 5 (33) | 4 (40) | 9 (36) |
| Arthralgia | 1 (7) | 3 (30) | 4 (16) |
| Nervous system disorders | 4 (27) | 3 (30) | 7 (28) |
| Hypoesthesia | 3 (20) | 1 (10) | 4 (16) |
| Respiratory, thoracic, and mediastinal disorders | 3 (20) | 2 (20) | 5 (20) |
| Oropharyngeal pain | 1 (7) | 2 (20) | 3 (12) |
|  | | | |
| MedDRA Version 23.0 | | | |
| Preferred terms of adverse events that occurred in more than or equal to three patients. | | | |

AE: adverse events; PT: preferred term; SOC: system organ class
