# Supplementary figures and images for: Phase III clinical trial of autologous CD34 + cell transplantation to accelerate fracture nonunion repair
Source: BMC Med. 2023 Oct 5;21:386. doi: 10.1186/s12916-023-03088-y (PMC10557317; doi:10.1186/s12916-023-03088-y)

**
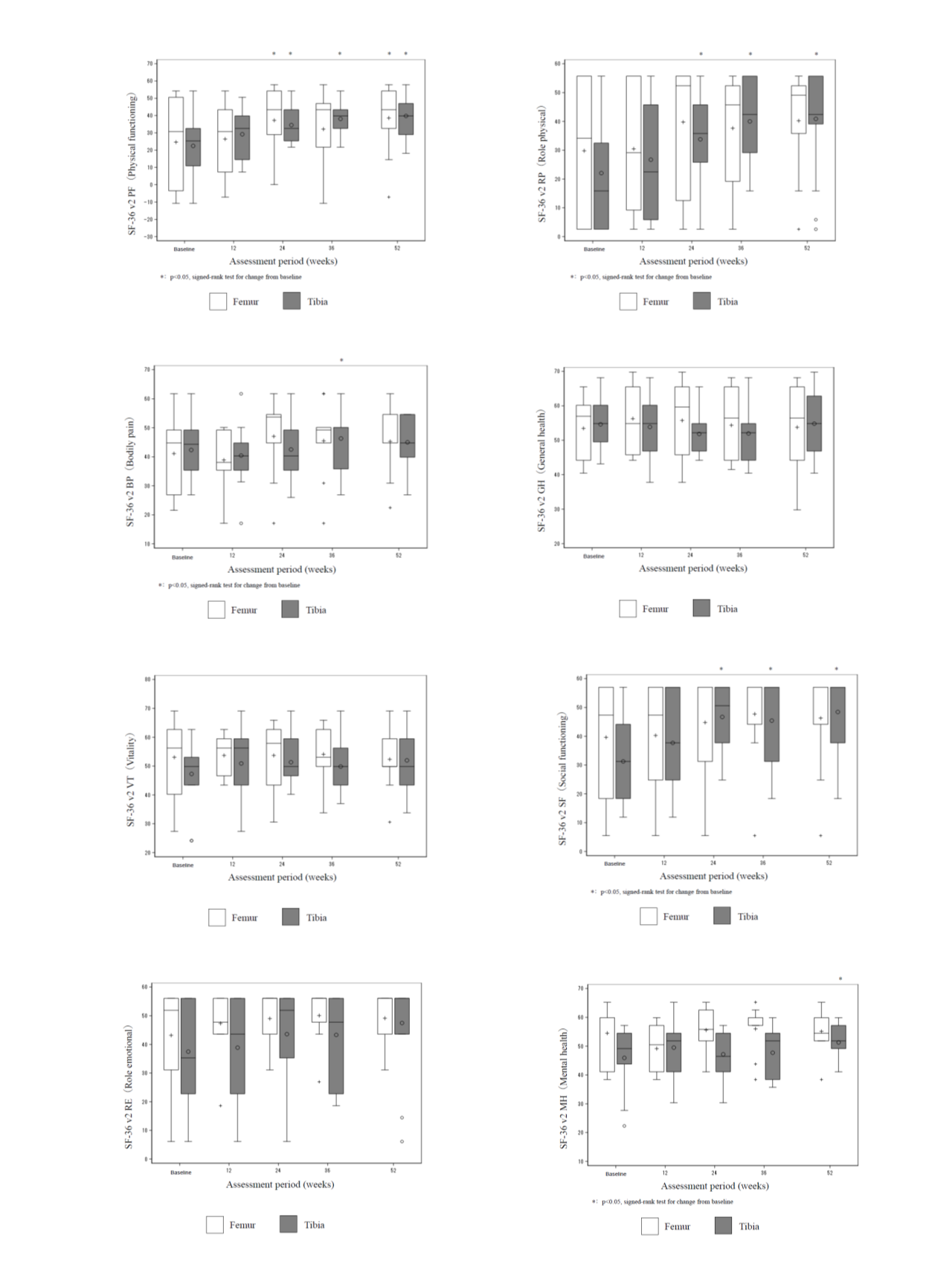
**

**b**

**d**

**h**

**g**

**e**

**f**

**c**

**a**

**Figure S1**

Supplement: Supplementary file 2 — Additional file 2: Figure S1. SF-36v2 scores of clinical trial patients treated with CD34+ cell transplantation assessed at different time-points. The horizontal axis shows the assessment period of 52 weeks. The vertical axis represents the SF-36v2 scores for a) physical functioning, b) physical role, c) bodily pain, d) general health, e) vitality, f) social functioning, g) emotional role, and h) mental health. The white boxes represent patients with femoral fracture nonunion, and gray boxes represent patients with tibial shaft fracture nonunion. Error bars represent standard deviation. [file 12916_2023_3088_MOESM2_ESM.docx]
